# SENOSI Confocal Microscopy: A New and Innovating Way to Detect Positive Margins in Non-Palpable Breast Cancer?

**DOI:** 10.3390/life14020204

**Published:** 2024-01-31

**Authors:** Deborah Wernly, Charles Beniere, Valerie Besse, Stephanie Seidler, Regine Lachat, Igor Letovanec, Daniela Huber, Colin Simonson

**Affiliations:** 1 Hôpital du Valais, 1951 Sion, Switzerland; charles.beniere@aurigen.ch (C.B.); valerie.besse@hopitalvs.ch (V.B.); stephanie.seidler@hopitalvs.ch (S.S.); regine.lachat@hopitalvs.ch (R.L.); igor.letovanec@hopitalvs.ch (I.L.); danielaemanuela.huber@hopitalvs.ch (D.H.); colin.simonson@hopitalvs.ch (C.S.); 2 Centre Hospitalier Universitaire Vaudois (CHUV), 1011 Lausanne, Switzerland; 3 Aurigen, Centre de Pathologie, 1004 Lausanne, Switzerland; 4 Department of Pediatrics, Gynecology and Obstetrics, Geneva University Hospitals, Boulevard de la Cluse 30, 1205 Geneva, Switzerland

**Keywords:** breast cancer, surgery, margins, confocal microscopy

## Abstract

In Switzerland, breast cancer is the leading cancer among women, with breast-conserving surgery (BCS) being the preferred treatment for small tumors. The margin status post-surgery is a critical predictor of local recurrence. Achieving negative margins remains a challenge, leading to re-excision in 20–30% of cases. Traditional methods like intraoperative examination palpation and radiography have limitations in assessing excised margins. This study introduces the Histolog^®^ Scanner, a confocal microscopy tool, as a potential solution. It provides real-time images of tissue architecture, allowing for rapid and accurate assessment of excised margins. Our research compared the Histolog^®^ Scanner with standard per-operative radiography in patients with non palpable breast cancer. Preliminary results indicate that the Histolog^®^ Scanner offers a reliable and time-efficient method for margin assessment, suggesting its potential for clinical integration.

## 1. Introduction

Breast cancer holds the position of the most frequently identified type of cancer and stands as the primary contributor to cancer-related fatalities among women on a global scale [1]. In Switzerland, breast cancer is the most frequent cancer in women, with 5300 new cases each year [2]. Breast-conserving surgery (BCS) is considered the preferred treatment choice for patients with small tumors, when feasible and when radiotherapy can be administered [3]. Local recurrence is influenced by the patient’s age, tumor size, grade, the presence of multifocal or multicentric disease, and margin status after the surgery [4]. The margin status is one of the strongest predictors of local recurrence among all these factors. Indeed, a positive margin is associated with more than a two-fold increase in local recurrence [5]. The optimal margin width in BCS for breast cancer has long been controversial [6]. Nowadays, consensus guidelines suggest that adequate margins for ductal carcinoma in situ (DCIS) should be ≥2 mm, whereas for invasive cancer, accompanied or not with DCIS, a negative margin is defined as “no ink on tumor” [7,8]. A positive margin means a re-excision a few weeks later, which can lead to anxiety, delays in adjuvant therapy, poor cosmetic results, and additional costs [9]. The rate of re-excision after a BCS to achieve negative margins varies between 20 and 30% [10,11,12].

Some studies showed that neither intraoperative examination palpation, radiography, or frozen section is reliable to assess the excised margin [13,14,15]. The surgeon can estimate the margin by gross palpation when the tumor is palpable. However, unfortunately, most early cancers are not palpable. That is why intraoperative specimen radiography is often performed to evaluate the radiological margins (the distance between the tumor’s image and the section margins) and the need for additional per-operative re-excision. Although this technique is the standard protocol in our institution, it has several drawbacks. It requires a radiographist and a radiologist on-site to acquire and read the images and it can be time-consuming, especially when additional ultrasound is requested. The sensitivity of surgical specimen radiographs ranges from 27% to 76% depending on the selected value for the margin [16]. Two main concerns are that a radiograph is a two-dimensional tool to evaluate a three-dimensional specimen, and that small tumor infiltrates cannot be identified confidently.

**Figure 1:**
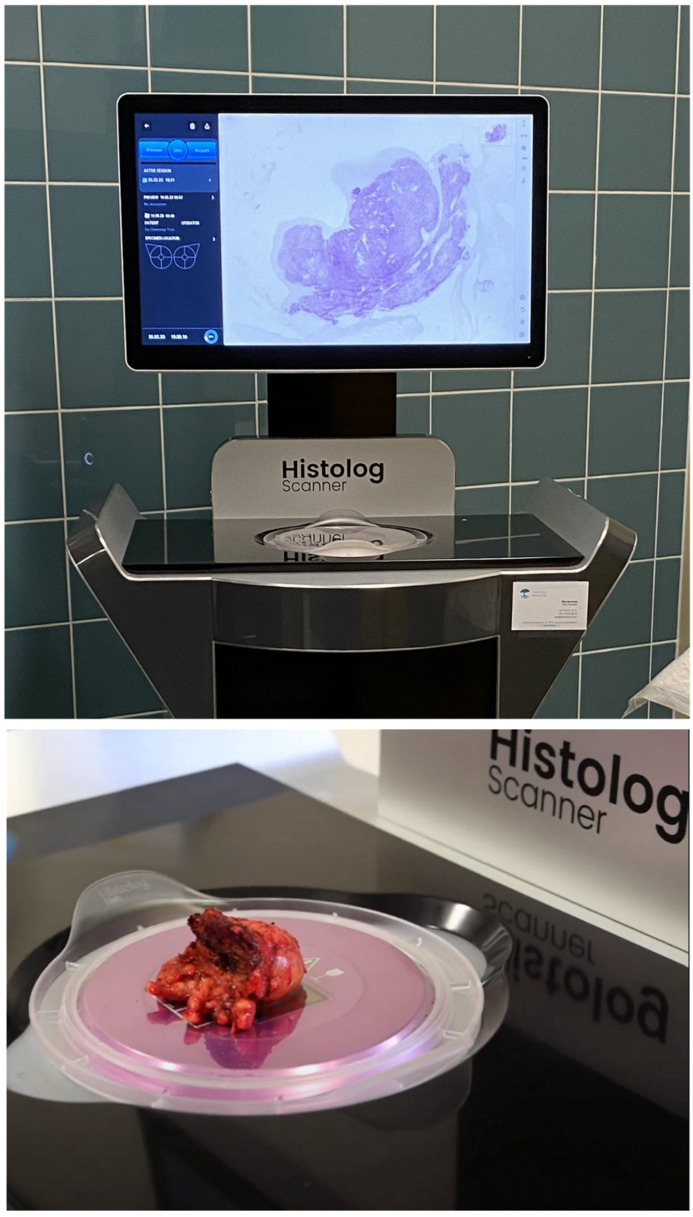
Histolog Scanner confocal imaging device (**top**) and full surgical specimen (unsliced) placed on the device for imaging (**bottom**).

Therefore, there is a demand for a method to evaluate excised margins in breast cancer that is not only more reliable but also more cost-effective in terms of reducing person-hours and operating room occupancy. Confocal microscopy has been described in the biomedical field as an effective method for imaging fresh tissue and the analyzed surface corresponds to the optical field of view of the microscope, ranging from 10 µm^2^ to 1 cm^2^ depending on the magnification setting. Confocal laser scanning microscopes (CLSMs) have been designed to tailor this technology for clinical applications by allowing an automated scan of several cm^2^ in a few minutes [17,18,19]. Confocal laser scanning microscopy has been shown to be a promising tool that provides real-time tissue architecture and morphology images for breast and skin tissue [20,21,22,23,24]. Recently, a CLSM has been made available for the medical imaging of large tissue specimens. With this device, sample preparation is rapid and simple. The piece of lumpectomy, freshly removed, is briefly stained and imaged within minutes, without the need for fixation or sectioning to reveal the microscopic morphology of the tissue, as seen in Figure 1 [25,26]. The pathologist, but also the surgeon, can interpret the images in the operating theater [20,26]. So far, confocal microscopy has shown promising results in evaluating the tissue morphology of a specimen and assessing the presence of cancer. In the field of breast cancer, two studies on 50 patients each revealed promising results with the Histolog^®^ Scanner (Samantree, Lausanne, Switzerland) on lumpectomies, indicating that the image performance allows the detection of breast cancer in fresh human tissue with accuracy and high reliability [21,25].

The present study aims to compare this new and innovative confocal laser microscopy scanner (the Histolog^®^ Scanner (HS)) with per-operative radiography, our hospital’s standard protocol (SoC) for patients with non-palpable breast cancer.

## 2. Materials and Methods

### 2.1. Population and Study Design

The recruitment process took place between May 2021 and April 2022, and 52 patients with non-palpable breast cancer of any histological type who underwent breast-conserving surgery after wire-guided preoperative marking were included.

Participants who were minors, breastfeeding, or pregnant, as well as those who had received previous treatment for breast cancer, such as chemotherapy, surgery, or radiotherapy, were excluded. The protocol was approved by the Canton de Vaud Ethics Committee (No. 2020-02357). The present study has an observational retrospective design. Its purpose is to realize the assessment of the images after surgery to ensure that this assessment has no impact on patient treatment as defined by the protocol approved by the Ethics Committee. The motivation of this study is to realize an initial feasibility study before engaging into future clinical demonstrations that may include performing interventions on patients.

### 2.2. Materials

The Histolog Scanner (SamanTree Medical, Lausanne, Switzerland) is a CE-IVD medical device designed to image large surgical specimens within the operating room. It is a confocal laser scanning microscope integrating a touch screen to operate the device and review the images on site (as shown in Figure 1). The resolution of the scanning process is 2 µm. The surgical specimen must be briefly stained with a fluorescent dye solution (Histolog Dip, SamanTree Medical, Switzerland) before imaging with the Histolog Scanner. The tissue fluorescence is excited by a 488 nm laser while the light above 500 nm in wavelength is collected. The images are displayed without additional post-processing in a hematoxylin and eosine (HE)-like digitally rendered virtual staining coloring. The device is ready to use a few seconds after being switched on, without the need for calibration or parameter setting. Full-resolution images are obtained within 50 s of acquisition to visualize thin details of tissue morphology up to the cell nuclei.

The Histolog Scanner allows for the imaging of unsliced specimens to visualize the whole surface of the specimen. This means that the full surgical specimen is imaged on the scanner “as it is”, without any sectioning (Figure 1). Having access to histological information in real time without any specimen slicing nor microscopy slide preparation necessary is exactly the innovation brought by this technique. All lateral margins from the full surgical specimen were imaged with this device as defined by the protocol without any prior selection.

### 2.3. Objectives

Our primary objective is to compare the performance (accuracy, sensitivity, sensibility) of confocal imaging of a fresh excision with intra-operative radiography to detect positive margins after breast lumpectomy.

Furthermore, we will also examine the following secondary objectives: a comparison of the time required for processing the lumpectomy specimen and evaluating the margins with both radiology and ultra-fast confocal imaging, and an assessment of the surgeon’s ability to interpret the confocal images compared to that of the pathologist.

### 2.4. Methods

**Figure 2:**
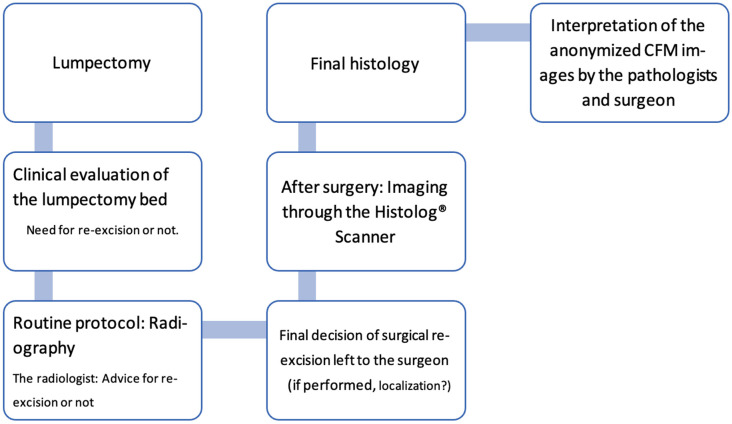
Diagram presenting the observational workflow implemented for the study.

SENOSI (which stands for the combination of ‘Senologie’ and ‘Sion’) is a prospective, monocentric, observational study method in which every excised lumpectomy specimen from all enrolled patients will be subjected intraoperatively to both confocal imaging and standard-of-care specimen radiography. The final pathology assessment is used as the study reference (Figure 2).

#### 2.4.1. Training in Histolog Images

In this study, neither the surgeons nor the pathologists had previous experience with interpreting confocal images. Initially, it was essential to acquaint them with the Histolog Scanner’s image content. To achieve this, they underwent a 2 h training session that involved studying reference materials. These materials showcased how breast cancer appears on the Histolog Scanner, featuring an extensive collection of 145 confocal images. This collection included 70 images of normal healthy tissue, along with 30 images of invasive ductal carcinoma (IDC), 25 of ductal carcinoma in situ (DCIS), and 20 of invasive lobular carcinoma (ILC). The training was designed to be comprehensive yet concise enough to fit within a 2 h window, aligning with the physicians’ workload.

#### 2.4.2. Standard of Care (SoC)

In our facility, the practice for lumpectomies involves uniformly excising tissue from the skin/subcutaneous layer down to the muscle layer. Consequently, we do not place significant emphasis on the superficial and deep margins. Post lumpectomy, the surgeon performs a standard clinical assessment of both the removed tissue and the surgical site. The excised tissue is then sent to the radiology department for standard processing. Radiographic examination of the lumpectomy tissue primarily focuses on the lateral margins: superior, inferior, lateral, and medial. Should radiography alone prove insufficient for a thorough margin evaluation, the radiologist may employ additional ultrasonography. The surgeon is then informed of the radiological findings and any suggestions for further excision, all within the timeframe of the ongoing surgical procedure. Ultimately, the surgeon decides whether to proceed with any additional excision.

#### 2.4.3. Histolog Imaging

After completion of the surgery, the lumpectomy specimen is prepared for imaging using the Histolog^®^ Scanner. The fresh surgical specimen is immersed in Histolog Dip solution for 10 s and then rinsed with NaCl 0.9% to remove any excess dye. The Histolog Dip contains a fluorescent agent that binds to negatively charged molecules such as nucleic acids and some proteins, allowing the visualization of cell nuclei and tissue morphology. All of the clinically relevant margins of the specimen are then imaged, producing coded high-resolution images.

Finally, the lumpectomy is sent to the pathology department for final histological analysis following standard-of-care procedures.

#### 2.4.4. Data Collection

Coded confocal microscopy images are interpreted blindly by the pathologist and the surgeon to evaluate the clinically useful margins (medial, lateral, inferior, and superior).

Evaluation of the margins assessed by gross palpation, radiography, and by confocal imaging, and the time requested by the surgeon for image acquisition, indication, and localization of cavity re-excision (if any is performed) are all reported.

### 2.5. Statistics

The data were collected from two sources: surgeons and pathologists. The data were analyzed using descriptive statistics, accuracy, sensitivity, specificity, positive predictive values (PPVs) and negative predictive values (NPVs). Sensitivity (true positive rate) measures the ability of a diagnostic test to correctly identify true positive cases (patients with the condition). It is calculated using the following formula: sensitivity = (true positives)/(true positives + false negatives). Specificity (true negative rate) measures the ability of a diagnostic test to correctly identify true negative cases (patients without the condition). It is calculated using the following formula: specificity = (true negatives)/(true negatives + false positives). Negative predictive value (NPV) assesses the probability that a patient with a negative test result truly does not have the condition. It is calculated using the following formula: NPV = (true negatives)/(true negatives + false negatives). NPV helps evaluate how reliable a negative test result is in ruling out the presence of the condition. Positive predictive value (PPV): PPV assesses the probability that a patient with a positive test result truly has the condition. It is calculated using the following formula: PPV = (true positives)/(true positives + false positives). PPV indicates the accuracy of a positive test result in confirming the presence of the condition.

The patient characteristics were tabulated using measures of empirical distributions such as the mean with standard deviation (SD) as well as 95% confidence intervals (CIs) depending on the level of measurement for continuous outcomes and the absolute and relative frequencies for categorical outcomes. McNemar’s chi-squared test was used to determine whether there is a significant difference between the performance of HLS and the SoC, and a paired t-test was performed to compare the mean utilization time between the two procedures. Statistical analyses were performed using R (Version 1.0.136–© 2009–2016 RStudio, Inc.).

## 3. Results

### 3.1. Cancer Patient Characteristics

**Figure 3:**
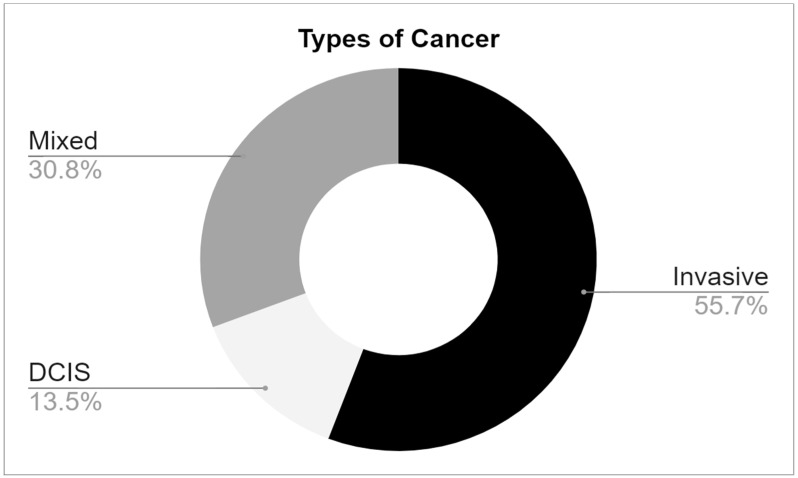
Distribution of breast cancer types identified by the final pathology assessment in study participants.

Fifty-two patients were recruited. The majority, 55.8%, had invasive carcinoma, while 33.8% had in situ carcinoma, and a minority, 13.5%, had a mixed form of the disease (Figure 3). Positive margins were observed in 21% of cases (11 patients) on the final pathological report of the primary excision, with an equal distribution among invasive and in situ cancers (Figure 3). 

#### Performance Analysis

**Figure 4:**
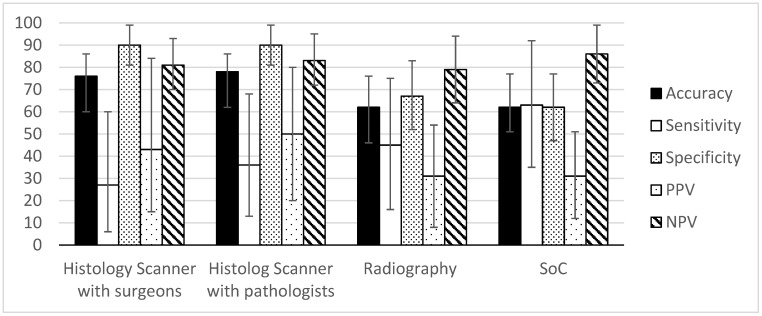
Detection performance and associated confidence intervals for individual techniques of the study.

**Figure 5:**
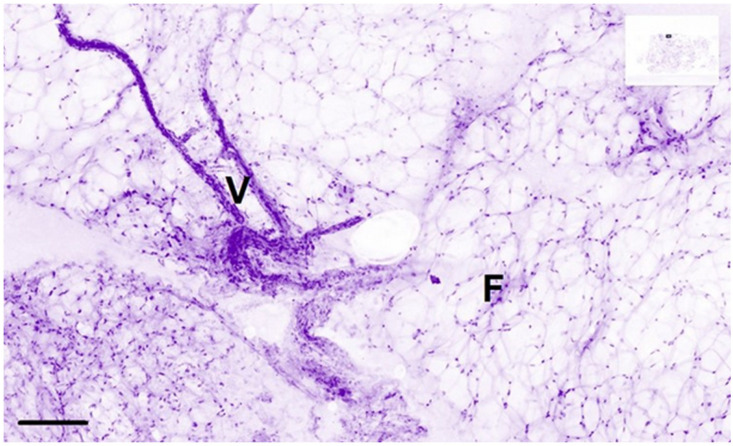
High-magnification Histolog images of a normal lumpectomy margin presenting healthy tissue with vessels (V) and fatty tissue (F). Scale bar is 100 µm.

**Figure 6:**
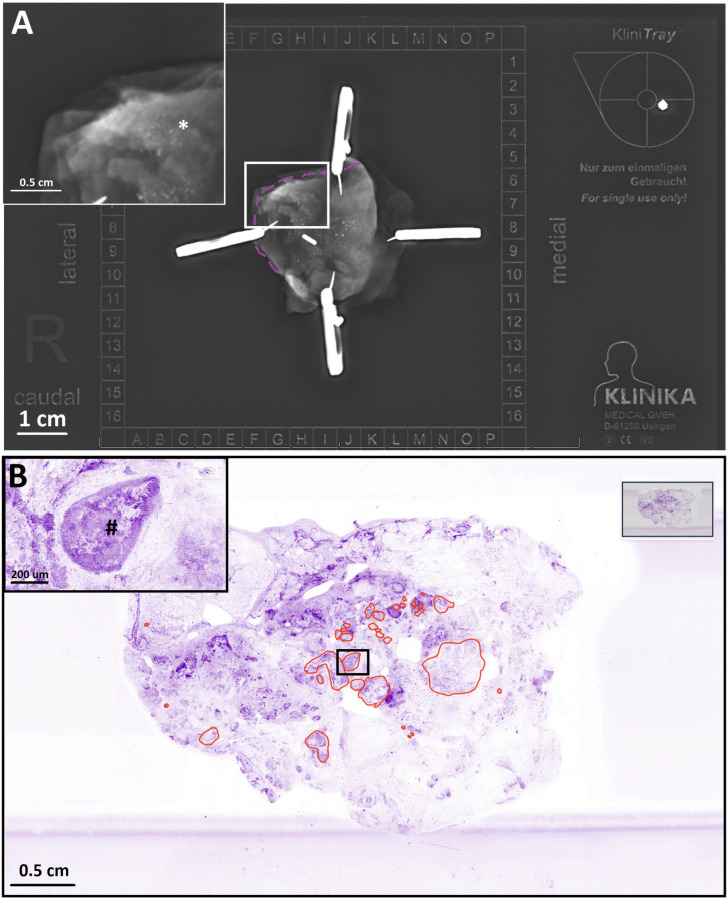
Study case with a DCIS-positive margin. (**A**) Radiography of the surgical specimen. Several microcalcifications can be seen in the center and in the upper-left quadrant. Some of them (*) are close to the surface of the margin (purple dashed line). The graphical insert shows the area within the white frame at higher magnification. (**B**) Low-magnification HS image of the surface of the cancer-positive margin corresponding to the specimen area with the purple dash line in the radiography figure. Areas delimited with red annotations are DCIS lesions. Graphical insert shows a DCIS lesion (#) at higher magnification, corresponding to the area within the back frame.

The pathologist and surgeon were able to recognize both normal and cancerous areas in HS images of lumpectomy margins (Figure 4, Figure 5 and Figure 6). The associated accuracy, PPV, and PPN values were higher than the values obtained by the radiologist, indicating a promising perspective for the technique in comparison to SoC techniques.

The analysis of the Histolog images by the surgeons had an accuracy of 76.47% (95% CI = 60–86%), sensitivity of 27.27% (95% CI = 6–60%), specificity of 90.00% (95% CI = 80.7–99.2%), PPV of 42.86% (95% CI = 15.3–84.6%), and NPV of 81.82% (95% CI = 70.4–93.2%) for breast cancer detection. For the pathologist, the accuracy was 78.43% (95% CI = 62–86%), sensitivity was 36.36% (95% CI = 13–68%), specificity was 90.00% (95% CI = 80.7–99.2%), PPV was 50.00% (95% CI = 20–80%), and NPV was 83.72% (95% CI = 72.6–94.7%) (Table 1).

In the course of the study, the radiography recommendation for each patient was noted in the Case Report Forms, enabling the extraction of radiography performances alone. It is relevant to compare the technique alone, as the Histolog Scanner images were analyzed blindly in an observational setting, and thus without other intraoperative inputs (see below). The recommendations of the radiologist based on radiography images alone had an accuracy of 62.22% (95% CI = 46–76%), sensitivity of 45.45% (95% CI = 16–74.8%), specificity of 67.65% (95% CI = 51.9–83.3%), PPV of 31.25% (95% CI = 8.5–53.9%), and NPV of 79.31% (95% CI = 64.5–94%) for breast cancer. As the intraoperative assessment is usually based on a combination of several techniques that may not be in agreement, surgeons decide to perform intraoperative recuts based on all of the available information such as radiography, palpation, visual inspection, and other factors (possibility to make a recut, risk-benefits) representing the true standard-of-care practice (SoC). The SoC practice had an accuracy of 62.75% (95% CI = 51–77%), sensitivity of 63.64% (95% CI = 35.2–92%), a specificity of 62.50% (95% CI = 47.4–77.5%), a PPV of 31.82% (95% CI = 12.3–51.2%), and an NPV of 86.21% (95% CI = 73.6–98.7%) (Table 1).

McNemar’s chi-squared test was performed to compare the performance of Histolog Scanner and standard-of-care. The analysis showed that there was a significant difference between the performance of the two methods for both the surgeons and pathologists (*p*-value = 0.0455 and 0.02474, respectively). The results suggest that the HS performed better than the standard-of-care for both the surgeons and pathologists. Overall, HS had the highest PPV (surgeons = 42.86%, pathology = 50%), indicating that when cancer lesions are detected, it is more likely to be correct than for radiography alone or in combination with intraoperative inputs (SoC). On the other hand, intraoperative inputs improved the NPV of radiography (radiography alone = 79.3%, SoC = 86.2%), indicating that the additional inputs gathered during surgery enable more reliability when ruling out disease. The HS NPV is in the range of the SoC; these data suggest that using the HS in combination with intraoperative inputs may further improve its performances, supporting its insertion into clinical routine.

### 3.2. Intraoperative Recuts

Intraoperative recuts were performed for 22/52 patients, including 15 recuts that were recommended by radiography. Out of these, 31% (7/22) were necessary, meaning positive margins on the lumpectomy specimen on final pathology. Despite intra-operative recuts, 3/7 had still a positive margin on the final pathology. Four out of seven intraoperative recuts enabled negative margins to be reached at the primary surgery, including two recuts recommended by radiography and two missed by radiography, but performed based on clinical impressions of the surgeon (Table 2).

**Figure 7:**
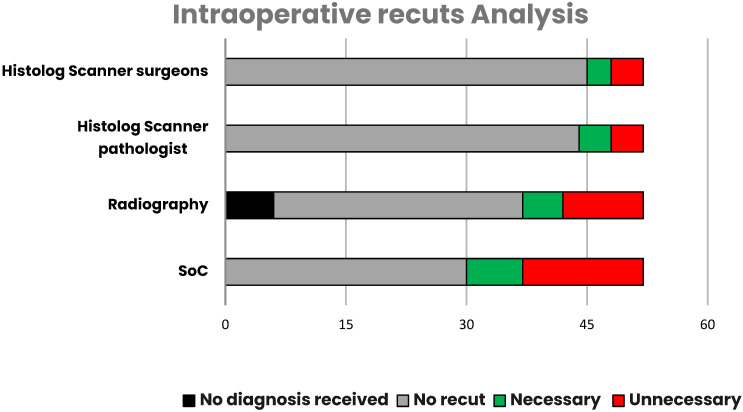
Bar plot displaying the number of intraoperative recuts recommended by individual techniques (radiography, HS surgeon, HS pathologist, and the standard-of-care (SoC)).

Overall, unnecessary recuts were performed in 29% of the patients with the SoC. On the other hand, the analyses of HS images by the surgeon and pathologist would have induced only 8% of unnecessary recuts. In addition, input from the radiologist was not received before the end of the surgery in 11.5% of the cases (6/52 patients), either because the specimen radiography was not performed, or the images could not be interpreted in time (Figure 7).

### 3.3. Timing Analysis

We conducted a comparison of the time required to evaluate margins using radiography and the Histolog Scanner. In the case of radiography, the timer was initiated when the lumpectomy was sent to the radiology department and stopped when the radiologist contacted the surgeon with a recommendation regarding the need for further recuts. On the other hand, for histological acquisition, the timer began when the lumpectomy was immersed in the solution and concluded when the images were obtained.

**Figure 8:**
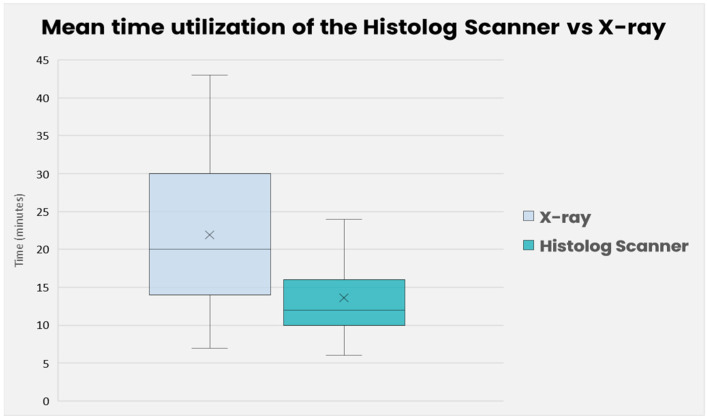
Mean time of utilization of the Histolog Scanner vs. radiography. The brackets, the cross (×), and the bar (-) represent the standard deviation, mean, and the medial of the collected values, respectively.

The difference in mean time between the radiography and HS was evaluated between the paired two samples for means calculation method. The mean time of evaluating margins with radiography was 21 min and 56 s (SD = 1.42), and with HS it was 13.44 min (SD = 0.65). The *t*-test showed a statistically significant difference between both means (t = 5.1726, *p* = 4.68 × 10^−6^). The analysis suggests that the HS had a significantly lower mean time than the standard-of-care (Figure 8).

## 4. Discussion

Enhancing the precision of intraoperative assessments for lumpectomy margins could allow us to achieve negative surgical margins in the primary surgery. Various techniques have been proposed to reduce the rate of cancer-positive margins and the need for subsequent re-operations in breast-conserving surgery (BCS). These techniques encompass the gross examination of the lumpectomy specimen, frozen sections, touch prep analysis, intraoperative specimen radiography, intraoperative ultrasound (US), and experimental tools [27,28,29]. In this study, we present the assessment of lumpectomy margins for breast cancer utilizing the Histolog Scanner, a medical device that offers morphological evaluation of large tissue specimens using the confocal scanning imaging approach. The magnification allowed with the HS corresponds roughly to a 10× microscopy lens, allowing a comprehensive understanding of the architecture of the tissue, as is commonly needed for intraoperative assessments. Nuclear details are hardly accessible at this magnification but such a level of detail is usually required only for the final pathology assessment. This method provides monochromic purple images that are similar to frozen section slides stained with Toluidine Blue. There is no need for freezing or slide preparation, and this is of particular interest for real-time assessments of breast tissue since its high content of fat makes frozen sections very difficult to obtain in a timely manner with this tissue. The device can be seamlessly integrated into the operating room, with rapid preparation and imaging times that align with the demands of the clinical workflow.

### 4.1. Performance Review

Assessment of HS images and radiography leads to the detection of positive margins in different patients. Therefore, the combination of the two methods allows for the detection of 9/11 cases of positive margins, thus increasing the detection rate to 81%. On the other hand, the use of the HS provides an efficient specificity of 90%, indicating that its use on patients is safe and will not induce unnecessary recuts, which is the initial purpose of BCS. These data indicate that the performance of our standard of care with per-operative radiography is expected to be enhanced by using the HS, which would encourage its integration into our clinical practice.

As mentioned above, out of 11 patients with intra-operative suspected-positive margins, only 7 of them had positive margins in the final histopathologic report. Indeed, negative margins were achieved by performing additional surgical recuts during the primary surgery in four out of seven cases. On these seven patients, the Histolog Scanner would have detected cancerous cells and avoided surgical resection in two patients, allowing an overall reduction of 29% in the positive margins.

Usually, intraoperative recuts are not analyzed due to the lack of time and resources. This additional assessment has been recently proposed by Togawa et al. with the Histolog Scanner thanks to its speed, ease of use, and high specificity [25]. In our study, four cases required justifiable resections by the surgeon due to positive margins on primary lumpectomy. However, these resections were deemed insufficient as the outer margins still tested positive. Therefore, the intraoperative assessment of recuts with the Histolog Scanner may further decrease the necessity for secondary surgeries, reducing costs and lessening the psychological and aesthetic impact on patients.

Additional training is anticipated to enhance the cancer detection rate significantly. The manufacturer is currently offering advanced training specifically for breast cancer, which, according to reports from other centers, may enhance the interpretation of confocal images, although these data remain unpublished. Furthermore, as Conversano et al. (2023) have discussed, the integration of Artificial Intelligence features in the analysis of confocal images is expected to not only improve but also standardize cancer detection in digital imagery. These advancements suggest a promising direction for improving overall performance in cancer detection and diagnosis [26].

### 4.2. Time and User Analysis

The assessment of margins through per-operative radiography takes up to 30% longer compared to using the Histolog Scanner, which is statistically significant. Moreover, it should be noted that in 11% of cases, radiography was unable to be performed or interpreted by the radiologist. This occurred due to the unavailability of the radiologist, radiology technician, or transporter, either during the noon period or towards the end of the day, highlighting the substantial organizational challenges of this technique.

Contrarily, the use of the HS for a period of 11 months has been performed without any technical issues and offers an easy user experience. The scanner is readily accessible for surgeons and offers independence in the interpretation of images during surgery.

### 4.3. Limitations

The study is subject to a few limitations, notably the limited sample size, which could affect the broader applicability of the findings. Being an observational study, its outcomes require validation via more extensive, prospective research. While the initial results of this feasibility study appear encouraging, further confirmation through studies involving a larger patient cohort is necessary, particularly to assess its effectiveness across less common subtypes of breast cancer.

Moreover, patients with previous treatment for breast cancer, such as chemotherapy, surgery, or radiotherapy, were excluded from the study because this type of patient was not present in the training material. Since such treatment can have some impact on the tissue histology, a dedicated study focused on these patients will have to be conducted in order to conclude the detection performance with this population.

Another limitation is the associated learning curve for interpreting images using the HS. This surely affects the accuracy of the results. Since the end of the patient enrollment, a deeper training material on breast features in confocal images has been made available from the manufacturer and from the literature, and we expect this increase the detection rate, especially for DCIS lesions [30].

Additionally, the utilization of electrocautery as part of the surgical technique in this study may have impacted the results due to the tissue retraction making it challenging to identify cancerous cells in images, as previously reported [21,25]. In the workflow of this observational study (Figure 2), the specimen was sent to radiology and imaged with the Histolog Scanner after the surgery. The consequence of this is a substantial time between the specimen excision and confocal imaging that results in an alteration of the specimen surface at a microscopic level, impeding the quality of Histolog images and therefore the detection rates by physicians. Unfortunately, this finding was not identified at the time of our study, but has been recently reported in a study that also included a round trip of the specimen between radiology and surgery prior to Histolog imaging [25]. Our suggestion is therefore to carry out confocal imaging prior to sending it to the radiology to ensure good image quality, as reported in another study that included an exclusively cold scalpel for specimen excision and rapid ultrasound assessment performed directly in the OR prior to Histolog imaging [21].

Concerning the time and user analysis, our study’s data analysis does not incorporate the duration of image analyses by the surgeons and pathologist. This consequently introduces an analysis bias. An interventional study would be warranted with intraoperative interpretation of images to determine if margin assessment using histology (performed by the surgeon), including acquisition and evaluation, is truly faster than sending the specimen to the radiology department for radiography and interpretation by the radiologist.

## 5. Conclusions

These findings indicate that the Histolog Scanner holds potential as an alternative or in combination to intra-operative radiography to detect positive margins in lumpectomies. Fast and precise, the Histolog Scanner has surely a place in clinical practice. Further studies with a larger sample size in an interventional setting are required, potentially including the intraoperative assessment of the recuts.

## Figures and Tables

**Table 1:** This table includes the accuracy value, sensitivity value, specificity value, positive predictive value (PPV), and negative predictive value (NPV) for the Histolog Scanner images reviewed by surgeons or pathology, radiography recommendation alone, or radiography recommendation in combination with other intraoperative inputs, considered here as the standard-of-care (SOC).

Medical Procedure	Accuracy	Sensitivity	Specificity	PPV	NPV
Histolog Scanner with surgeons	76.47%	27.27%	90.00%	42.86%	81.82%
Histolog Scanner with pathologists	78.43%	36.36%	90.00%	50.00%	83.72%
Radiography	62.22%	45.45%	67.65%	31.25%	79.31%
SoC	62.75%	63.64%	62.50%	31.82%	86.21%

**Table 2:** This table presents the recommendations to perform intraoperative recuts for HS images reviewed by surgeons, pathologist, and specimen radiography alone. The SoC row corresponds to the actual intraoperative recuts that were performed by surgeons based on clinical impressions and specimen radiography when available. * Feedback from the radiology department was not available for 6 patients.

	No Recut	Recut		Total Recut
		Necessary	Unnecessary	
Histolog Scanner Surgeon	45	3	4	7
Histolog Scanner Pathologist	44	4	4	8
Specimen Radiography *	37	5	10	15
SoC	30	7	15	22

## Data Availability

The datasets generated and analysed during the current study are available from the corresponding author on reasonable request.
